# Cytochrome *bd* Displays Significant Quinol Peroxidase Activity

**DOI:** 10.1038/srep27631

**Published:** 2016-06-09

**Authors:** Sinan Al-Attar, Yuanjie Yu, Martijn Pinkse, Jo Hoeser, Thorsten Friedrich, Dirk Bald, Simon de Vries

**Affiliations:** 1 Department of Biotechnology, Delft University of Technology, The Netherlands; 2 Institut für Biochemie, Albert-Ludwigs-Universität Freiburg, Albertstr 21, 79014 Freiburg i. Br., Germany; 3 Department of Molecular Cell Biology, AIMMS, Faculty of Earth- and Life Sciences, VU University Amsterdam, Amsterdam, The Netherlands

## Abstract

Cytochrome *bd* is a prokaryotic terminal oxidase that catalyses the electrogenic reduction of oxygen to water using ubiquinol as electron donor. Cytochrome *bd* is a tri-haem integral membrane enzyme carrying a low-spin haem *b*_558_, and two high-spin haems: *b*_595_ and *d*. Here we show that besides its oxidase activity, cytochrome *bd* from *Escherichia coli* is a genuine quinol peroxidase (QPO) that reduces hydrogen peroxide to water. The highly active and pure enzyme preparation used in this study did not display the catalase activity recently reported for *E. coli* cytochrome *bd*. To our knowledge, cytochrome *bd* is the first membrane-bound quinol peroxidase detected in *E. coli*. The observation that cytochrome *bd* is a quinol peroxidase, can provide a biochemical basis for its role in detoxification of hydrogen peroxide and may explain the frequent findings reported in the literature that indicate increased sensitivity to hydrogen peroxide and decreased virulence in mutants that lack the enzyme.

Cytochrome *bd* is an integral membrane terminal oxidase that uses ubiquinol as the physiological electron donor for catalysing the reduction of molecular oxygen to water^1^,^2^,^3^,^4^. This strictly prokaryotic oxidase is found in many bacterial pathogens^5^,^6^,^7^,^8^,^9^,^10^,^11^,^12^,^13^,^14^ and contributes to the formation of the proton motive force by vectorial charge transfer without actual proton pumping^15^,^16^,^17^,^18^,^19^. Protons (H^+^_cyto_) are taken up at the cytoplasmic side of the membrane for water formation whereas quinol (QH_2_) oxidation leads to proton (H^+^_peri_) release at the periplasmic side (Eq. 1).


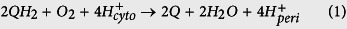


The bioenergetic efficiency of cytochrome *bd* is half that of the oxygen-reducing cytochrome oxidases, which in addition to consuming chemical protons also pump protons across the membrane (reviewed in^20^).

Cytochrome *bd* is a tri-haem protein carrying haem *b*_*558*_ which is ligated by His186 and Met393 (*Escherichia coli* numbering), haem *b*_595_ ligated by His19 and Glu99, and haem *d* ligated by Glu445^21^. Haems *b*_595_ and *d* are proposed to constitute a functional binuclear site, similar to the binuclear haem-Cu_B_ site in haem-copper oxidases where the oxygen chemistry takes place^20^,^22^,^23^,^24^,^25^,^26^,^27^ (but see^21^). An important mechanistic feature in both classes of enzymes is that the reduction of oxygen occurs in a concerted 4-electron redox reaction preventing the formation of reactive oxygen species (ROS): superoxide (O_2_^−^), hydrogen peroxide (H_2_O_2_) and the hydroxyl radical^28^.

ROS are produced endogenously when molecular oxygen is partially reduced to superoxide and H_2_O_2_ by redox enzymes, especially flavoenzymes, including the respiratory chain Complex I^29^,^30^,^31^,^32^,^33^,^34^. Two superoxide anions dismutate to H_2_O_2_ and O_2_ in the cell either spontaneously or catalysed enzymatically by superoxide dismutase (SOD). When H_2_O_2_ is reduced by cellular Fe^2+^ through Fenton chemistry, hydroxyl radicals are produced leading to a wide spectrum of damage to biological molecules^35^,^36^. In addition to lipids and DNA, protein targets of ROS, which lead to enzyme inactivation, include solvent-exposed Fe-S clusters of dehydratases among which aconitases and fumarases and the Isc system responsible for Fe-S cluster synthesis^37^,^38^. Cells not only have to cope with endogenous ROS. Microorganisms must also detoxify ROS produced extracellularly by competing microorganisms and in the case of pathogenic microorganisms by host immune systems^39^,^40^,^41^.

In order to protect themselves from oxidative damage, prokaryotes express different ROS scavenging enzymes and employ low-molecular weight agents such as ascorbate and glutathione^36^,^42^,^43^. In addition to SOD, *E. coli* synthesizes a number of specific cytoplasmic H_2_O_2_-scavenging enzymes: the catalases KatG and KatE^36^; NADH-dependent alkyl hydroperoxide reductase (Ahp)^44^, glutathione peroxidase (GPX)^45^,^46^ and thiol peroxidase^47^,^48^. In aerobically growing *E. coli* cells, the main H_2_O_2_ scavengers are KatG, KatE and Ahp^42^.

Cytochrome *bd* has been proposed to confer protection to oxygen-sensitive enzymes and to help protect the cell from nitrosative and ROS stresses^7^,^49^,^50^,^51^,^52^,^53^,^54^,^55^. It was shown that cytochrome *bd* knockouts were highly sensitive to hydrogen peroxide and showed increased levels of endogenous ROS^6^,^54^,^56^,^57^, including ROS resulting from antibiotic-induced stress^58^,^59^. Cytochrome *bd* knockouts in *Mycobacteria* were highly susceptible to drugs acting on oxidative phosphorylation^58^,^60^,^61^. In addition, numerous studies concerning pathogenic bacteria indicated that lack of a functioning cytochrome *bd* severely compromises virulence and intracellular viability^6^,^10^,^11^,^12^,^13^,^51^,^52^,^58^,^62^,^63^.

Collectively, these studies indicate that cytochrome *bd* can play a role in scavenging exogenous H_2_O_2_ produced e.g. during infection in a manner similar to periplasmic catalases/peroxidases or SOD, which have been proposed as virulence factors in highly pathogenic bacterial strains among which *E. coli* O157:H7 and several other species^64^,^65^,^66^,^67^,^68^.

Two recent studies have suggested that cytochrome *bd* from *E. coli* is endowed with very low guaiacol peroxidase activity^69^ and a significant catalase activity^70^, proposed to explain the protective phenotype of the enzyme *in vivo*.

In the present study, we aimed to investigate the *in vitro* activity of a highly purified preparation of cytochrome *bd* towards hydrogen peroxide. Mass spectrometry showed the presence of a third subunit, CydX. We further show that cytochrome *bd* has quinol peroxidase (QPO) activity and lacks catalase activity. We discuss how the newly discovered QPO activity of cytochrome *bd* can contribute to detoxification of exogenous hydrogen peroxide, therefore potentially contributing to the virulence of pathogenic microorganisms.

## Materials and Methods

### Materials

Decylubiquinone, Coenzyme Q_0_ (UQ-0), 30% hydrogen peroxide (concentration determined using ***ε***_240nm_ 44 M^−1^ cm^−1 ^71), bovine liver catalase and lauroyl sarcosine were purchased from Sigma-Aldrich. 1,4-Dithiothreitol (DDT) was from GERBU. 2-n-Heptyl-4-hydroxyquinoline N-oxide (HQNO) was from Enzo Life Sciences (New York). Lauryl maltoside (LM) was purchased from Affymetrix.

### Protein preparation and activity assays

Expression and purification of the wild type cytochrome *bd* was performed using a *cydABX* pACYC177 overexpression vector as described earlier^28^. For production of the His_6_-tagged protein, the vector was modified by addition of six histidine triplets (CACCATCACCACCATCAC) at the 3′-end of *cydA* (C-terminal His_6_-tag). Overexpression of the His_6_-tagged protein and membrane isolation were done as in^28^. The protein was purified over a HisTrap Nickel column (GE Healthcare) eluting at ~ 0.3 M imidazole (0.02–0.5 M imidazole gradient). The protein was further purified using a Superdex 200 gelfiltration column (GE Healthcare). The haem *d* content was determined spectrophotometrically from the dithionite-reduced minus as isolated difference spectrum using ***ε***_630–650nm_ = 24 mM^−1^ cm^−1^^72^. The protein content was determined with the BCA assay (Uptima, Interchim). The purity of the protein was assessed based on the haem *d*/protein ratio we found (9.26 μmol haem *d*/g protein) which corresponds to ~97% using a molar weight of 105.5 kDa for His_6_-CydABX. Polarographic oxidase activity measurements and lack of catalase activity were performed and confirmed in two groups either using a home-built setup with a Clark-type oxygen electrode^73^ (Group Simon de Vries) or an Oxygraph+ Clark-type oxygen electrode from Hansatech (Group Thorsten Friedrich). Determination of quinol peroxidation rates was conducted inside a Coy anaerobic chamber equipped with an Avantes DH-2000 spectrophotometer. Due to the high 278-nm absorbance at high quinol/quinone concentrations, the reaction progress was monitored at 260 nm rather than 278 nm. The extinction coefficient ***ε***_260nm_ = 6.23 mM^−1^ cm^−1^ was determined from the UV spectrum of decylubiquinone based on ***ε***_278nm_ = 12.7 mM^−1^ cm^−1^. The reactions were performed in the standard buffer: 50 mM MOPS, 100 mM NaCl, 0.1% LM, pH 7 unless otherwise noted. Aliquots of nitric oxide (NO) were added from a NO-saturated (2 mM) aqueous solution.

Igor Pro version 6.1 (Wavemetrics) was used for data analysis and creating graphs.

### Analysis of the steady-state kinetics

Initial QPO rates were determined for varying H_2_O_2_ concentrations while keeping the decylubiquinol (dQH_2_) concentration constant and *vice versa*. The rates were simulated using the model for a Ping-Pong Bi Bi reaction mechanism according to:


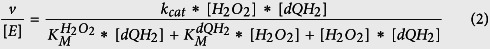


Herein *k*_cat_ represents the maximal turnover number (s^−1^) and [*E*] the enzyme concentration.

### Determination of the reaction stoichiometry

At different time intervals, the QPO reaction (50 μM H_2_O_2_, 200 μM dQH_2_ and 60 nM cytochrome *bd*) was quenched with 200 mM HCl and incubated for 2 minutes prior to neutralization with 200 mM NaOH. The concentration of H_2_O_2_ at each time point was determined using the Amplex Red/Horseradish peroxidase H_2_O_2_ assay kit (Invitrogen) using ***ε***_571nm_ = 58 mM^−1^ cm^−1^ for resorufin. The dQH_2_ concentration was determined in the same experiment from the absorbance change at 260 nm as described above.

### Determination of catalase activity in membranes

The catalase activity in membranes was determined by following the oxygen production (see above) in standard buffer without detergent at different H_2_O_2_ concentration. To test whether the catalase activity was membrane-associated, the membranes of *E. coli* overexpressing cytochrome *bd* were washed by first diluting the membrane suspension (1:13) in standard buffer without detergent. The diluted suspension was sonicated (10 min in a Biorupter sonicator from Diagenode at maximum intensity) to disrupt possible membrane vesicles containing cytosolic proteins. The sonicated membrane suspension was centrifuged for 1 h at 300,000 g for membrane recovery. The membrane pellet was resuspended in buffer prior to the polarographic assay. The dilution/sonication procedure was repeated (second wash) using the product from the first step and the polarographic assay was performed again.

### Tandem MS analysis and identification of CydX

Purified His-tagged cytochrome *bd* was loaded on a Native-PAGE, the protein band of interest was excised from the gel and subjected to in gel proteolytic digestion using either trypsin, chymotrypsin or GluC (enzyme: protein ratios ~1:15–1:20 (w/w) in 25 mM ammonium bicarbonate, pH 8.1) overnight at 37 °C. Prior to digestion, cysteines were reduced with dithiothreitol (DTT) in ammonium bicarbonate for 30 min, followed by alkylation with iodoacetamide in ammonium bicarbonate in the dark for 45 min. In-gel digests were separated and analyzed on EASY-nLC 1000 system directly coupled to a Q Exactive Plus mass spectrometer (Thermo, Bremen, Germany). Peptides were separated on a reversed-phase column (Acclaim PepMap, 50 μm × 150 mm, 2 μm, 100 Å, Thermo, Bremen, Germany). The gradient was from 100% Solvent A (0.1% formic acid in water) to 60% solvent B (acetonitrile) in 25 min. at a flow rate of 500 nl/min. The column effluent was directly electrosprayed in the ESI source of the mass spectrometer using a nano-ESI emitter (Nano-bore emitter, Thermo, Bremen, Germany). MS data was acquired in the positive ion mode using a data-dependent top10 analysis method. Full scan spectra were acquired in the *m/z* range 400–1200 at a resolution of 70.000, a target value of 3e6 and a maximum injection time of 100 msec. HCD fragmentation events were dynamically triggered at an underfill ratio of 5%. Isolation of precursor ions was done with a window of 2,5 amu, a target value of 2e5 and maximum injection time of 50 msec. Normalized collision energy of 27 eV was used and fragment ions were acquired at a resolution of 17.500 with *m/z* 100 as first mass. The raw data was processed with Proteome Discoverer 1.4 (Thermo, Bremen, Germany) and spectra were matched against the Uniprot protein database using mascot. Search parameters used were; 5 ppm for precursor mass, 0.02 Da for fragment ions, taxonomy restrain *E. coli*, carbamidomethylcysteine as fixed modification and oxidized methionine as variable modification and no cleavage enzyme was specified. CydX from *E. coli* consists of 37 amino acid residues (1-MWYFAWILGTLLACSFGVITALALEHVESGKAGQEDI-37).

### Analytical chromatography

To verify the monodispersity of the pure cytochrome *bd*, 500 μg of the enzyme was loaded onto a gel filtration column (Superose 6 10/300 gl, GE Healtcare) equilibrated with the standard buffer (VE2001 GPC solvent/sample module, Viscotek). The UV absorbance at 280 nm (UV Detector 2600, Viscotek) as well as the refractive index and the right angle light scattering were monitored during the run (TDA 305 triple detector array, Viscotek).

## Results

### The cytochrome *bd* preparation is highly pure and contains the CydX subunit

**Figure 1:**
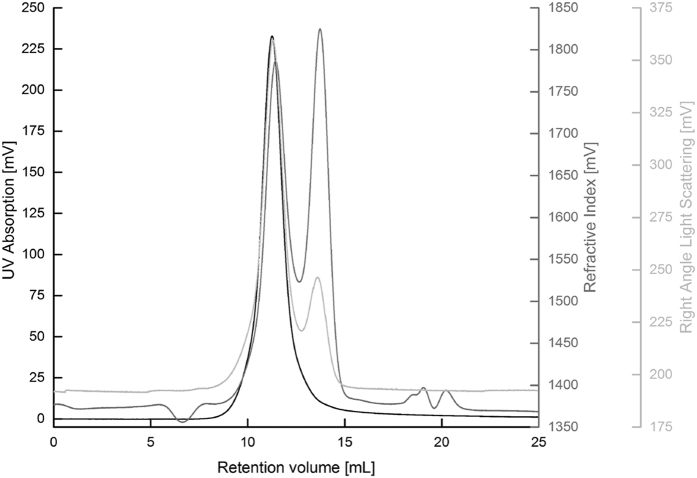
Analytical chromatography of purified cytochrome *bd*. Purified cytochrome *bd* was subjected to analytical gel filtration chromatography. UV absorption at 280 nm is shown in black, refractive index and right angle light scattering are shown in gray and light gray, respectively. A monodisperse protein peak was detected at an elution volume of 11.3 mL, corresponding to a mass of approximately 470 kDa. A second non-protein peak was detected at 13.7 mL, corresponding to a mass of approximately 70 kDa.

Using decylubiquinol (dQH_2_) as electron donor, the purified cytochrome *bd* displayed a turnover number of 185 ± 15 dQH_2_ s^−1^ consistent with the value for the wild-type enzyme^72^ indicating that the His-tag did not interfere with the activity of the enzyme. Cytochrome *bd* has long been considered a hetero-dimer throughout literature^1^,^2^,^3^,^20^. However, recent mutational studies in *E. coli, Brucella abortus* and *Shewanella oneidensis* suggested that the small protein, CydX (37, 64 and 38 amino acids in *E. coli, B. abortus* and *S. oneidensis*, resp.) is important for assembly, stability and activity of cytochrome *bd in vivo* and *in vitro*^63^,^74^,^75^,^76^. The presence of CydX has also been confirmed in purified cytochrome *bd*^75^. To confirm the presence of CydX in our preparation we performed a mass-spectrometric analysis. Using trypsin, chymotrypsin and Glu-C to cleave the protein we detected the peptides 23-ALEHVESGKAGQEDI-37, 29-SGKAGQEDI-37 and 23-ALEHVESGKAGQEDI-37, respectively, unequivocally confirming the presence of CydX in our preparation. To verify the monodispersity of the purified cytochrome *bd*, the enzyme was subjected to analytical chromatography (Fig. 1). The UV absorption showed a single peak, corresponding to the mass of the cytochrome *bd* tetramer including the LM micelle (approx. 480 kDa). Refractive index and right angle light scattering exhibited a second peak (approx. 70 kDa) corresponding to the average size of an empty LM micelle. Based on the haem *d*/protein ratio (see Materials and Methods) the protein purity was approximated as ~97%. These results show that the isolated cytochrome *bd* is pure, active and complete.

### Cytochrome *bd* is a quinol peroxidase

**Figure 2:**
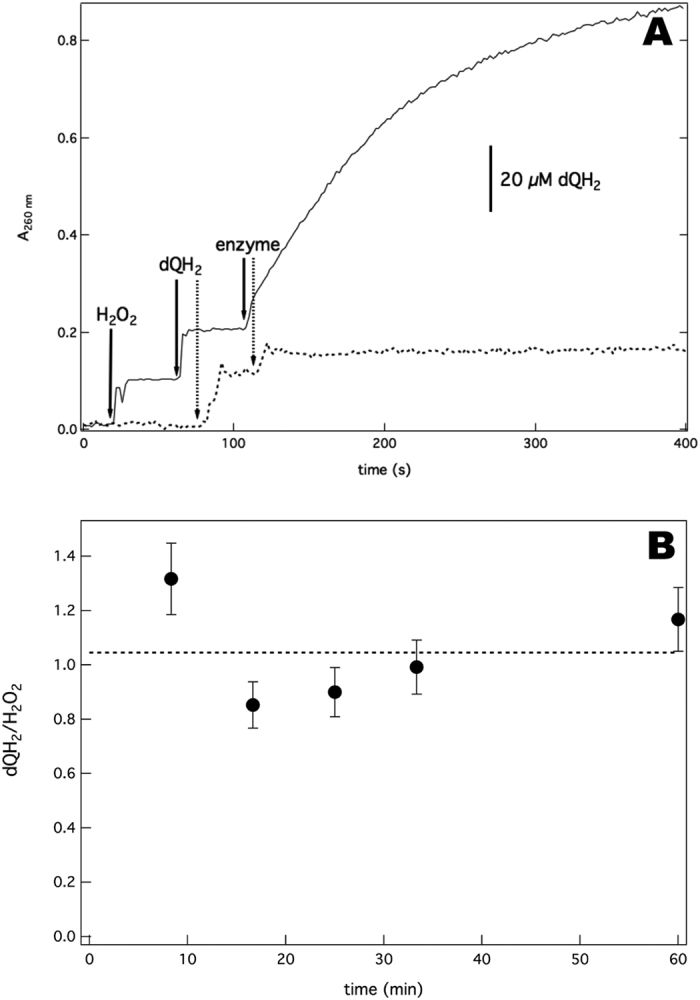
Cytochrome *bd* has quinol peroxidase activity. (**A**) The QPO reaction catalysed by cytochrome *bd* is monitored as dQH_2_ oxidation (260 nm). The dotted trace represents a control experiment where only the enzyme and dQH_2_ are added showing a lack of background activity and inferring that the system is anaerobic. Upon addition of H_2_O_2_, dQH_2_ is oxidized (solid trace). The reaction buffer contained 120 μM dQH_2_ and 23 nM cytochrome *bd* with (solid trace) or without (dotted trace) 6 mM H_2_O_2_. Solid and dotted arrows indicate the time of the additions corresponding to the solid and dotted traces, respectively. (**B**) The dQH_2_/H_2_O_2_ ratio of the QPO reaction catalysed by cytochrome *bd*. The average dQH_2_/H_2_O_2_ ratio was determined at 1.05 ± 0.19 by analyzing the reaction buffer at different time intervals during the reaction. The average ratio is consistent with the peroxidase reaction (Eq. 3). The results are presented as means ± SD of duplicates (n = 2).

We tested whether cytochrome *bd* could function as a peroxidase with its natural oxidase substrate, ubiquinol. Reduced decylubiquinone was used as replacement for the natural ubiquinol-8 in *E. coli*^72^ and its oxidation was followed spectrophotometrically in the presence of H_2_O_2_ (Eq. 3). Experiments were carried-out strictly anaerobically to prevent interference between the oxidase and peroxidase reactions. Cytochrome *bd* was found to catalyse the oxidation of dQH_2_ in the presence of H_2_O_2_ (Fig. 2A). To confirm that the oxidation of dQH_2_ is due to dQH_2_:H_2_O_2_ oxidoreduction, i.e. QPO activity, we measured the amounts of both H_2_O_2_ and dQH_2_ consumed in time in order to determine the reaction stoichiometry. Figure 2B shows the calculated ratios of dQH_2_/H_2_O_2_, which average to 1.05 ± 0.19. This is consistent with the 1:1 stoichiometry predicted for a genuine QPO reaction (Eq. 3).


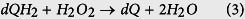


**Figure 3:**
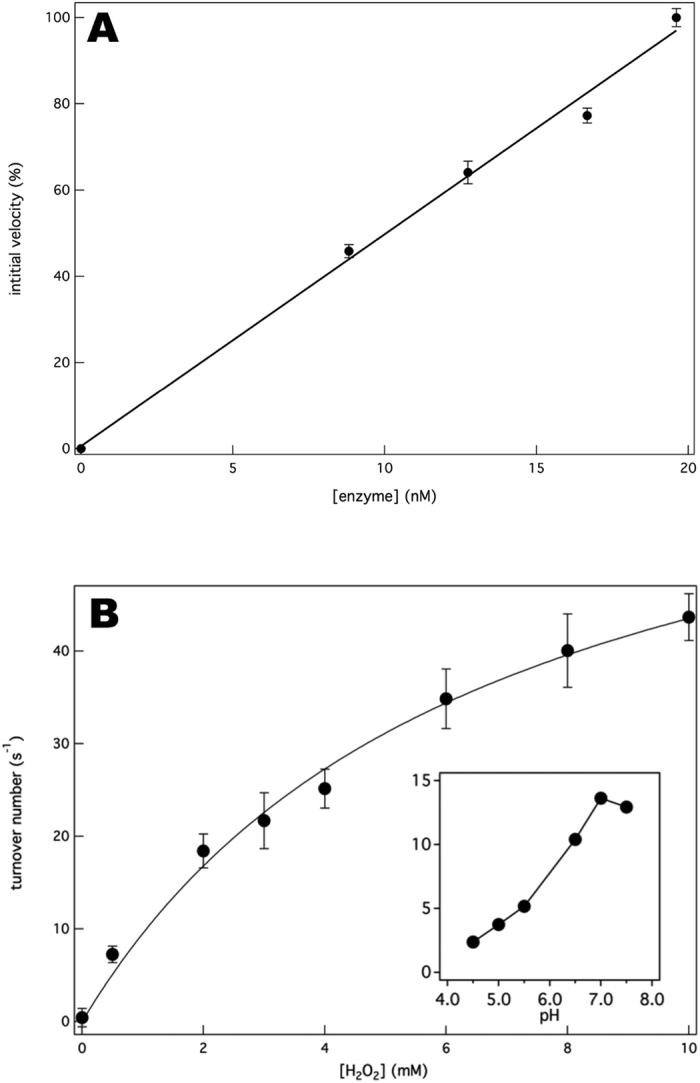
Kinetics of quinol peroxidase activity by cytochrome *bd*. (**A**) The proportional relation between the initial rate of quinol-peroxide reduction and cytochrome *bd* concentration. The QPO initial rates were measured in standard buffer in the presence of 120 μM dQH_2_ and 1 mM H_2_O_2_ at room temperature. (**B**) The QPO activity of cytochrome *bd* as function of the H_2_O_2_ concentration showing saturation kinetics. Initial rates are expressed as turnover number (mol dQH_2_/mol enzyme/s). The data were fitted to the Michaelis-Menten equation (lines). The fitting parameters (apparent V_max_ and K_M_ values) were V_max_ = 75 ± 4.5 s^−1^ and K_M_ = 6.6 ± 1.1 mM. The inset shows the pH-dependence of the QPO reactions at 1 mM H_2_O_2_. The assays were performed in the presence of 120 μM dQH_2_ and 23 nM cytochrome *bd*. The results are presented as means ± SD of duplicates (n = 2). The inset shows single measurements.

To investigate the steady-state kinetics of the QPO reaction, the initial peroxidation rates were measured at different enzyme, H_2_O_2_ and dQH_2_ concentrations. The plot of initial rate versus the amount of enzyme shows a linear relationship (Fig. 3A). The K_M_ values for H_2_O_2_ and dQH_2_ were determined at 6.6 ± 1.1 mM and 72 ± 20 μM, respectively, with the latter being similar to the K_M_ (dQH_2_) of 85 ± 5 μM^72^ of the oxidase reaction (Fig. 3B). The maximal QPO *k*_cat_ calculated according to Eq. 2 was 101 ± 10 H_2_O_2_ s^−1^ yielding a specificity constant *k*_cat_/*K*_M_ (H_2_O_2_) = 1.5 10^4^ M^−1^ s^−1^. The QPO pH-dependence profile (inset Fig. 3B) is similar to that of the oxidase reaction^77^, with the highest activity at around pH 7. However, the oxidase reaction is completely inhibited at pH values lower than 5.5^77^ whereas at this pH the QPO reaction retains ~1/3 of its maximal value at pH 7.0.

### The QPO reaction is inhibited by oxidase inhibitors

**Figure 4:**
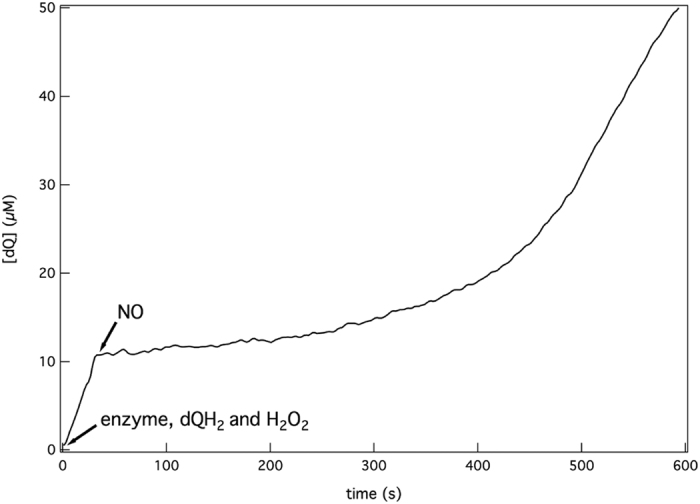
Inhibition of quinol peroxidase activity by nitric oxide. Reversible inhibition of the QPO reaction by NO was monitored spectrophotometrically. After addition of 6 μM NO, the reaction is inhibited promptly but resumes due to disappearance of NO. The QPO reaction was started by addition of 200 μM dQH_2_ and 10 mM H_2_O_2_ to 9 nM of cytochrome *bd* at room temperature.

NO which mainly binds haem *d*^27^, is a reversible inhibitor of the oxidase reaction^78^. Interestingly, our data show that also the QPO reaction is inhibited by NO as well (Fig. 4). Upon addition of 6 μM NO, dQH_2_ oxidation was drastically decreased. The inhibition was reversible as the activity slowly restored (Fig. 4), likely due to slow reaction between NO and dQH_2_ which was observed in a separate experiment (22 nM s^−1^ NO at 100 μM NO and 100 μM dQH_2_, data not shown). We did not detect a reaction between NO and H_2_O_2_ or any quinol:NO reductase activity, in agreement with others^78^. We also found that titration of the QPO and oxidase activities with HQNO shows that 50% inhibition is obtained at ~10–15 μM for both reactions.

### Cytochrome *bd* does not show catalase activity

**Figure 5:**
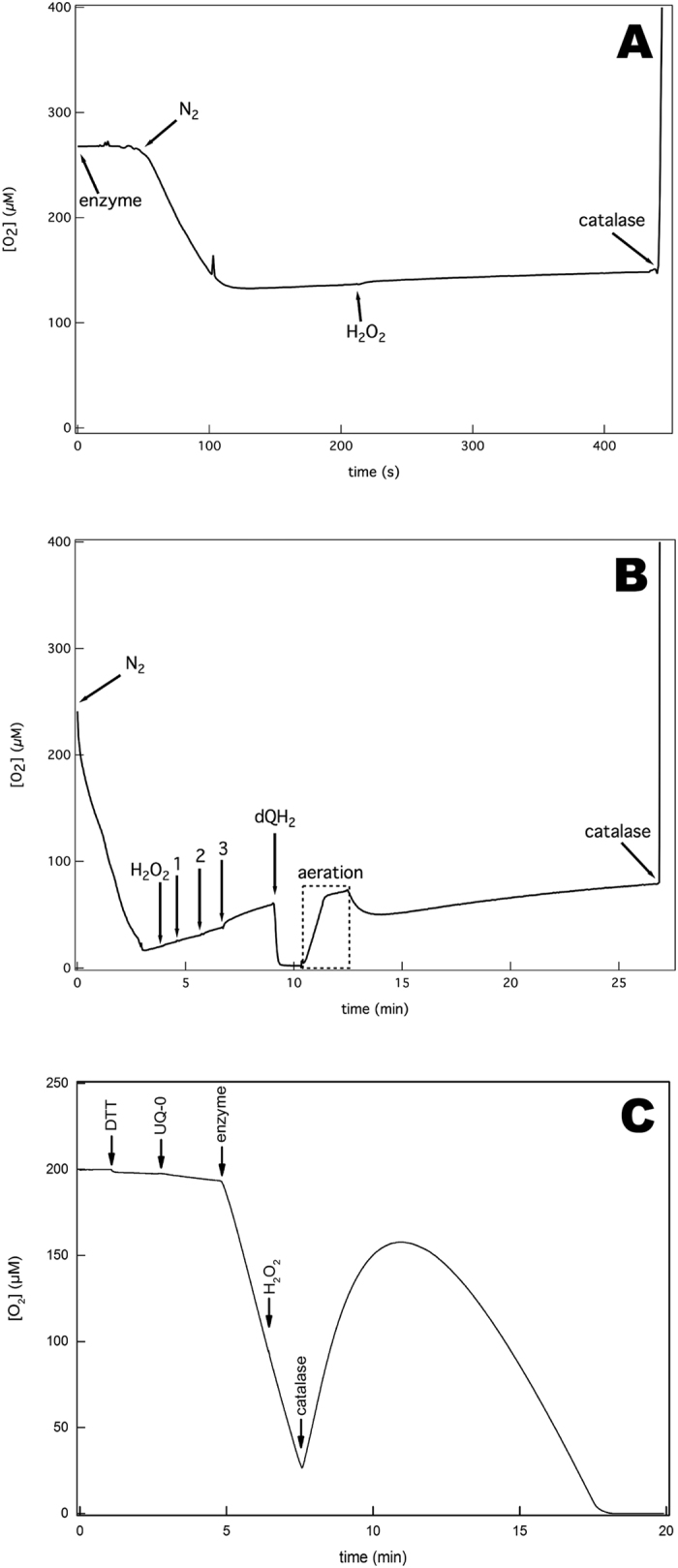
Lack of catalase activity of purified cytochrome *bd* in non- and mid-turnover conditions. (**A**) Oxygen measurement in the presence of cytochrome *bd* and H_2_O_2_ shows that the enzyme does not have catalase activity. The enzyme (125 nM) in standard buffer was first purged with nitrogen gas (N_2_) to lower the oxygen concentration to ~130 μM. The addition of 1 mM H_2_O_2_ did not show any generation of oxygen indicating the lack of catalase activity. As a positive control, 1 μM of catalase was added resulting in a rapid increase in oxygen concentration. Due to oxygen leakage into the measuring chamber, a slow background increase in oxygen concentration is observed. (**B**) Test for catalase activity by cytochrome *bd* using buffer and detergent reported in^70^. Lack of catalase activity of cytochrome *bd* determined polarographically. The buffer was 50 mM KPi, 0.1 mM EDTA, 0.05% LS, pH 7.0 the same as in^70^. The buffer was purged with nitrogen gas (N_2_) to lower the oxygen concentration prior to addition of 1 mM H_2_O_2_. Then, cytochrome *bd* was added successively indicated by numbered arrows: 1, 0.075 μM; 2, 0.225 μM and 3, 1 μM (accumulative concentrations). No catalase activity was detected after any of these additions. When 200 μM dQH_2_ was added a rapid decrease in oxygen concentration is observed due to the oxidase activity of cytochrome *bd*. (**C**) Polarographic test for catalase activity during turnover as previously reported in^70^. Oxidase turnover was started, by consecutively adding 10 mM DTT, 50 μM UQ-0 and 100 nM enzyme to the standard buffer. During turnover 1 mM of H_2_O_2_ was added to the reaction and a decrease in oxidase activity of 9 ± 2% could be observed. Oxygen formation was only observed after adding 20 nM catalase.

**Figure 6:**
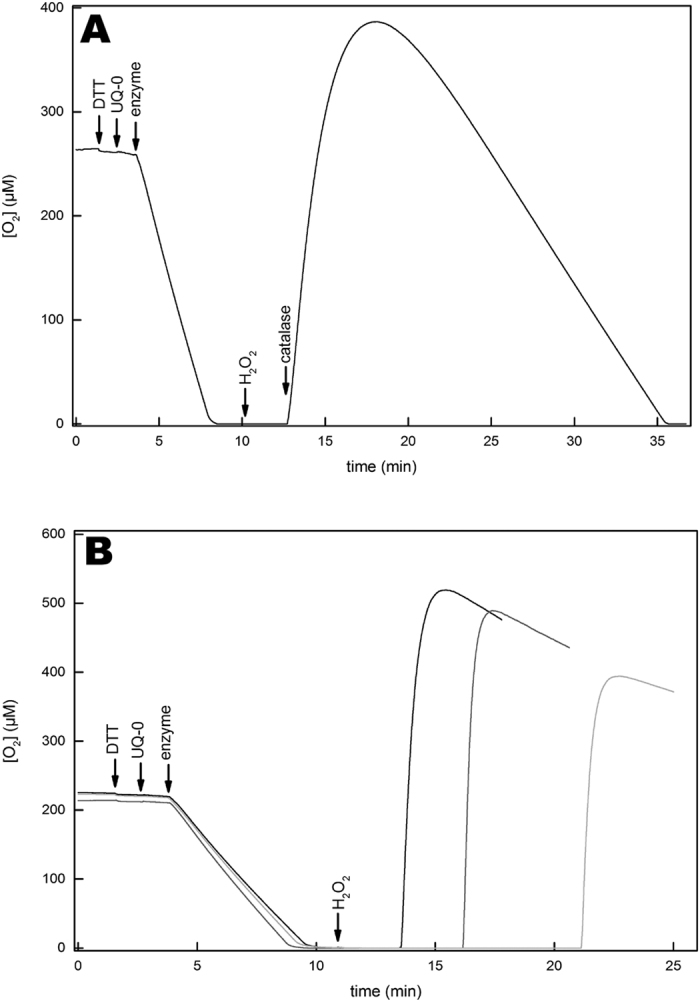
Lack of catalase activity of purified cytochrome *bd* in post-turnover conditions. Polarographic test for catalase activity after achieving anoxia through cytochrome *bd* oxidase turnover as in^70^. (**A**) Oxidase turnover was started, by consecutively adding 10 mM DTT, 50 μM UQ-0 and 100 nM enzyme to the standard buffer. 90 seconds after reaching anoxia, 1 mM of H_2_O_2_ was added to the reaction and no increase in oxygen could be observed. 150 seconds after peroxide addition, 20 nM catalase were added and formation of oxygen was observed. (**B**) Identical reaction parameters as in (**A**) were used. The reaction mixture was incubated for 2.5 (black), 5 (gray) and 10 minutes (light gray) after addition of 1 mM H_2_O_2_. After incubation, 100 nM of catalase were added and formation of different quantities of oxygen was observed.

Catalases produce oxygen and water from hydrogen peroxide (Eq. 4) allowing the detection of their activity polarographically using a Clark-type oxygen sensor. Recently it was reported that cytochrome *bd* from *E. coli* had catalase activity^70^. We tested our pure cytochrome *bd* preparation polarographically in standard buffer (Fig. 5A) and in the buffer (50 mM KP_i_ + 0.1 mM EDTA + 0.05% N-lauroylsarcosine, pH 7.0) used in ref. 70 (Fig. 5B). Even at enzyme concentrations as high as 1 μM cytochrome *bd* (Fig. 5B), catalase activity was absent. In Fig. 5C we show our attempt to reproduce the mid-turnover catalase activity measurement shown in ref. 70. Albeit we noticed a decrease of oxidase activity of 9 ± 2% upon addition of 1 mM H_2_O_2_ to the assay during turnover (Fig. 5C), we were not able to detect catalase activity under neither of these assay conditions. The lack of post-turnover catalase activity shown in Fig. 3 in ref. 70 was confirmed using similar reaction parameters (Fig. 6A). Interestingly, we observed that the quantity of released oxygen upon catalase addition is dependent of the incubation time of the enzyme with H_2_O_2_ (Fig. 6B). This supports the conclusion that H_2_O_2_ is consumed during the incubation process, but no oxygen is released, i.e. due to the QPO activity (Eq. 3).


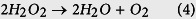


**Figure 7:**
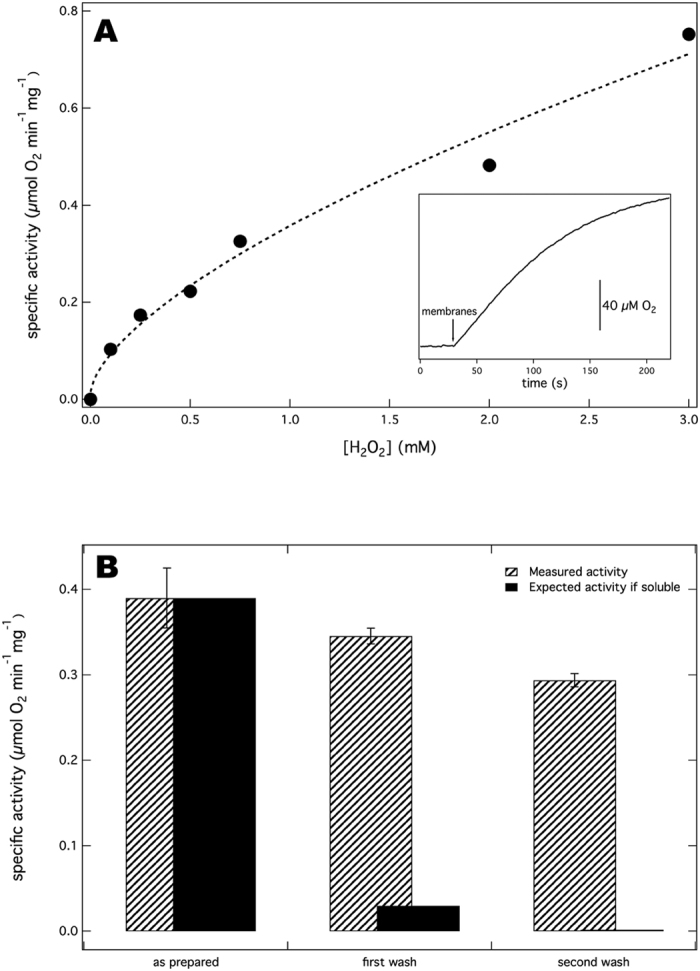
Catalase activity in an *E. coli* membrane fraction. (**A**) The dependence of the catalase activity of isolated *E. coli* membranes on H_2_O_2_ concentration. Membranes were added to H_2_O_2_ containing standard buffer. The dashed line is a non-hyperbolic power law fit: Specific activity = y_0_ + A * [H2O2]^n^. where y_0_ = 0.015, A = 0.34 and n = 0.65. The inset is a representative activity trace that shows oxygen formation in the presence of 0.25 mM H_2_O_2_. (**B**) The catalase activity is membrane-associated. A weak catalase activity measured at 1 mM H_2_O_2_ was observed in the membranes of *E. coli*. The bulk of the catalase activity (striped bars) was resistant to washing/sonication cycles (See Materials and Methods) indicating that the activity is membrane-associated. A theoretical activity profile (solid bars) is shown representing the expected remaining catalase activity for a soluble entity (7.7% and 0.60% remaining activity after the first and second washing steps, respectively). The results are presented as means ± SD of duplicates (n = 2).

We hypothesized that the catalase activity detected by the authors in ref. 70 might be due to impurities in their enzyme preparation. Therefore, isolated membranes from *E. coli* that overexpress cytochrome *bd* were assayed for catalase activity. Interestingly, the membranes did show a weak catalase activity (Fig. 7). It is notable that the relation between activity and H_2_O_2_ concentration (Fig. 7A) is very similar to that presented in the inset of Fig. 1 in ref. 70. The catalase activity profile is biphasic and non-hyperbolic, showing quite a sharp increase below ~0.2 mM H_2_O_2_ and levelling off at higher H_2_O_2_ concentrations unlike canonical catalases that show a linear relation at millimolar H_2_O_2_ concentrations^79^,^80^,^81^. These data suggest that the catalase activity reported in ref. 70 is due to an impurity in the cytochrome *bd* preparation, although we cannot rule out the possibility that the catalase activity may be dependent on the experimental conditions chosen for protein expression and purification.

To determine if the catalase activity we found in the membrane suspension is membrane-associated and not a cytosolic entity, the activity was measured after washing and sonicating the membranes in buffer containing no detergent (see Materials and Methods). The weak catalase activity decreased slightly after each washing step (Fig. 7B, striped bars), which is prescribed to inactivation and loss of material during the washing procedure. The results show that this catalase activity is resistant to washing suggesting it is membrane-associated. The catalases (KatG and KatE) in *E. coli* are soluble proteins and to our knowledge, no membrane-bound catalases have been reported in *E. coli*. Our results show the presence of an unknown membrane-associated catalase activity in *E. coli*. This hitherto unidentified catalase activity was not further investigated in this study.

## Discussion

The purpose of this study was to investigate the *in vitro* activity of cytochrome *bd* with hydrogen peroxide to highlight its potential anti-ROS activity *in vivo*. We have demonstrated here that cytochrome *bd* from *E. coli* is a bifunctional enzyme equipped with quinol-linked oxygen and H_2_O_2_ reduction activities. In addition, we have shown that the QPO reaction is inhibited by HQNO and NO similar to the oxidase reaction, which suggests a similar involvement of the haem centres and the quinol-binding site in both the oxidase and QPO reactions in respect to electron transfer and catalysis. Under the conditions employed in this study, the data showed that cytochrome *bd* does not function as a catalase. However, we did detect a membrane-associated catalase activity in isolated *E. coli* membranes not documented before that showed an unusual relation between activity and H_2_O_2_ concentration.

Quinol peroxidation is quite rare in prokaryotes. Besides cytochrome *bd*, another QPO was found in the human pathogen *Aggregatibacter actinomycetemcomitans.* This enzyme is a tri-haem *c* membrane-bound protein with ~43% sequence identity with bacterial cytochrome *c* peroxidases but less than 13% with cytochrome *bd*^82^,^83^. Inhibition of *Ac*QPO correlated to decreased pathogenicity of *A. actinomycetemcomitans*^83^, a phenotype typical for cytochrome *bd* mutants (see below)*. E. coli* contains a homologue of *Ac*QPO (YhjA^82^) predicted to be a cytochrome *c* peroxidase^43^. YhjA was also tested for QPO activity and was found negative^82^. To our knowledge, cytochrome *bd* is the first quinol peroxidase characterized in *E. coli*.

The QPO activity of cytochrome *bd* demonstrated could provide direct biochemical underpinning for understanding some phenotypes displayed by organisms with non-functional cytochrome *bd*. For example *E. coli* with disrupted cytochrome *bd* accumulated temperature-sensitive growth defects, which could be reverted by exogenous addition of reducing agents as well as SOD and catalase suggesting that increased ROS concentrations (induced at higher temperatures) can be counteracted by the peroxidative cytochrome *bd* activity^84^. The localization of cytochrome *bd* in the membrane, suggests that the enzyme can reduce exogenous H_2_O_2_ and is therefore functionally differentiated from Ahp, KatG and KatE that scavenge intracellular H_2_O_2_^32^,^85^,^86^,^87^,^88^.

As described in the Introduction^6^,^10^,^11^,^12^,^13^,^51^,^52^,^58^,^62^,^63^,^89^, many examples indicate that pathogenic bacteria that lack cytochrome *bd* activity display compromised virulence and viability. A striking example is provided by the *in vivo* anti-ROS activity of cytochrome *bd* in the gram-negative pathogen *B. abortus*^6^. *B. abortus* devoid of a functional cytochrome *bd* had severely compromised survival in murine spleens, but in *trans* over-expression of SOD, catalase or cytochrome *bd* complemented this phenotype showing that H_2_O_2_ accumulation is the main phenotype induced by lack of cytochrome *bd* activity^6^. Consistent with this finding, *Staphylococcus aureus* increases cytochrome *bd* expression 8–9 fold upon addition of 10 mM H_2_O_2_^90^ and *M. tuberculosis* with over-expressed cytochrome *bd* showed increased resistance to H_2_O_2_^91^, whereas a cytochrome *bd* knockout in this strain resulted in decreased survival in the mammalian host^10^. The predicted localization of the cytochrome *bd* active site at the periplasmic side of the cytoplasmic membrane may testify to its protective function mainly against environmentally produced H_2_O_2_ and against H_2_O_2_ produced in the phagocyte oxidative burst experienced by pathogenic bacteria residing in human macrophages. The finding that the oxidase reaction is completely inhibited at pH values lower than 5.5^77^ but the QPO reaction of cytochrome *bd* is not, may be relevant to its role in combatting the phagocyte oxidative burst in view of the low pH in the phagocyte^92^. It would be important to test cytochrome *bd* from pathogenic bacterial strains for QPO activity, and to evaluate the contribution of the QPO for survival in the host.

In summary, our finding that cytochrome *bd* exhibits QPO activity demonstrates that this respiratory complex can serve as a detoxifying enzyme.

In addition to indirectly decreasing the rate of intracellular ROS production via its oxidase reaction, cytochrome *bd* can also actively metabolize and detoxify hydrogen peroxide. As such, the very catalytic properties of cytochrome *bd* may explain how the enzyme can act as general virulence factor, which operates in concert with other virulence factors enhancing pathogenicity.

## Additional Information

**How to cite this article**: Al-Attar, S. *et al*. Cytochrome *bd* Displays Significant Quinol Peroxidase Activity. *Sci. Rep.* **6**, 27631; doi: 10.1038/srep27631 (2016).
